# Modern technology: lowering the radiation dose for lung cancer screening

**DOI:** 10.1186/1470-7330-14-S1-P39

**Published:** 2014-10-09

**Authors:** Luba Frank, Emmanuel Christodoulou, Ella Kazerooni

**Affiliations:** 1 University of Michigan, Ann Arbor, MI, USA

## Purpose

This review aims to present advances of modern technology that can be applied to lower the radiation risk for lung cancer screening.

## Background

Lung cancer remains one of the deadliest cancers with late detection and high mortality rates. Results of the NLST trial published in 2011 demonstrated a 20% decrease in lung cancer mortality in the CT arm compared to the radiography arm. One of the biggest concerns of wide implementation of lung cancer screening by CT, is radiation risk and radiation-induced cancer.

We present various techniques, from tube current modulation to low dose and ultra low dose CT with adapative statistical iterative reconstruction (ASIR) and model based iterative reconstruction (MBIR), that introduce much lower radiation dose, while remain diagnostic for lung cancer screening.

We will also present our model of radiation risk estimation based on lower radiation doses used in modern studies.

## Summary

This review will present newer currently available techniques for radiation risk reduction and our model of radiation risk estimation in comparison with available older models, based on calculations of radiation doses on population of atomic bomb survivors.

